# Mr. Chamberlain at the Criterion

**Published:** 1904-07-09

**Authors:** 


### Mr. Chamberlain at the Criterion.

Few more eloquent addresses have ever been delivered by any public men, or upon any public question, than those of Mr. Balfour and Mr. Chamberlain on the occasion of the dinner given to the latter statesman on the last day of June at the Criterion Restaurant. The dinner was given by the Royal Institute of Public Health in recognition of Mr. Chamberlain's services to preventive and tropical medicine during his tenure of office as Colonial Secretary; and it not only afforded to Professor William Smith, M.D., the President of the Institute, an opportunity of giving some outline of these services, but to the guest of the evening, and, after him, to the Prime Minister, equally valuable opportunities of asserting the position which sanitary questions must for the future occupy in the estimation of all statesmen. Professor Smith was able to declare that, after having rendered priceless service to the general sanitation of Birmingham, Mr. Chamberlain had distinguished himself at the Colonial Office by advocating and assisting the foundation of the London School of Tropical Medicine, an institution which, in its turn, had led up to the foundation of the Liverpool School, and had directed to the subject an attention which had already been extraordinarily successful in dealing with the problems which it was called upon to face. It might, indeed, be said of Mr. Chamberlain, in the well-remembered words of one of the greatest of English martyrs, that he had lighted a candle which would never be suffered to go out. Mr. Chamberlain, in his reply, recalled the speech made by Mr. Disraeli in the early seventies, in which he spoke of the importance of sanitary reform as the foundation of every other, and which was at the time made the subject of some ridicule, in which the speaker admitted that he himself had joined. "None of us," he went on to say, "are likely to ridicule such a statement now"; and he proceeded to declare his conviction that sanitary reform is not unworthy of the attention of the highest statesmanship. Without it social reform is but an empty phrase. The housing of the poor, the attempts to prevent the physical deterioration of the race, depend or are founded upon sanitary reform. Preventible disease is at this moment a great agent for filling our workhouses, for raising our taxes, for weakening the fibre of our people, for preventing us from competing successfully in the eternal struggle for existence which shall go on as long as the world shall last. In war it is even more important than in peace; for preventible disease destroys more soldiers than the swords or the bullets of the enemy, and cripples the efficiency of the administration of the army. Mr. Chamberlain then gave a glowing account of the proceedings of Englishmen as pioneers and colonists in all countries, and dwelt upon the claim they had for protection against the strange diseases to the often unknown causes of which they were exposed. It was this view of the case which enlisted his warmest sympathies, and it was to the efforts of such men as Sir Patrick Manson, Major Ross, Professor Haffkine, and others that his attention had been mainly directed. He was conscious that, after everything possible had been done for the colonial empire by statesmen, it might be that some of the almost unknown students who were working in laboratories in London or Liverpool were after all doing more than any statesman, however high his position, could ever hope to accomplish. Mr. Balfour, who followed in response to the toast of "Preventive Medicine and the State," endorsed to the full what the guest of the evening had said, and pointed out that only by improved sanitation could we hope to preserve the race from deterioration in the changing social circumstances of the day, and in the relative increase in the number of town dwellers which those circumstances produced. On the other hand, it was only by the same agency, and by research into the nature of tropical disease, that we could preserve the lives of the many Englishmen who were required for the superintendence, either civilian or military, of many of our dominions beyond the sea. To turn our great tropical colonies into places where the white man could live in reasonable health and comfort would be to add, directly and indirectly, incalculable gains to the commercial future and the industrial prosperity of this country. It was this work, among much other, in which Mr. Chamberlain had been engaged, and it would have as great an effect upon the permanent colonial empire of this country as anything which had ever been done by a single man or a single body of men.

